# Effects of molecular weight fraction on antioxidation capacity of rice protein hydrolysates

**DOI:** 10.1038/s41598-022-14314-7

**Published:** 2023-03-01

**Authors:** Hui-Ju Chen, Fan-Jhen Dai, Cheng-You Chen, Siao-Ling Fan, Ji-Hong Zheng, Chi-Fai Chau, Yung-Sheng Lin, Chin-Shuh Chen

**Affiliations:** 1 Department of Food Science and Biotechnology, National Chung Hsing University, Taichung, 402204 Taiwan; 2 Healthmate Co., Ltd, Changhua, 500016 Taiwan; 3 Ph.D. Program in Materials and Chemical Engineering, National United University, Miaoli, 360302 Taiwan; 4 Department of Chemical Engineering, National United University, Miaoli, 360302 Taiwan; 5 Institute of Food Safety and Health Risk Assessment, National Yang Ming Chiao Tung University, Taipei, 112304 Taiwan

**Keywords:** Biochemistry, Biological techniques

## Abstract

Rice protein was used as a starting material to provide rice protein hydrolysates (RPH) through enzyme-assisted extraction. RPH was further fractionated using ultrafiltration membrane (UF) and classified by molecular weight (MW; MW < 1 kDa, MW 1–10 kDa, and MW > 10 kDa). Peptides with MW < 1 kDa possessed superior antioxidant properties (*p* < 0.05). Therefore, UF demonstrated great efficacy in selectively separating antioxidant peptides. A Pearson correlation analysis revealed that the total phenolic concentration was correlated with oxygen radical absorbance capacity (ORAC; r = 0.999, *p* < 0.05). Amino acid contents had negative correlations with the scavenging activity (specifically, IC50) of 1,1-diphenyl-2-picrylhydrazyl and 2,2'-azino-bis (3-ethylbenzothiazoline-6-sulfonic acid) radicals (r =  − 0.986 to − 1.000). Reducing power was related to aromatic amino acid contents (r = 0.997, *p* < 0.05). In this study, enzymatic hydrolysis was discovered to be an effective method of extracting and isolating natural antioxidant proteins from broken rice, thus preserving the nutritional quality of rice and making those proteins more accessible in future applications.

## Introduction

Plant protein provides almost 65% of human protein intake worldwide and is a valuable ingredient in many applications. The increasing appeal of plant protein is attributed to the increase in the global population, the increase in living expenses, the limited supply of animal protein, and people becoming more conscious toward their food intake^1^. Protein has consistently been among the top ten factors driving the current healthy food market. It is viewed as a wellspring of energy and essential amino acids and as a functional ingredient that is beneficial to health^2^. The vast majority of known peptides are obtained from costly protein frameworks (including those in food), which makes their application generally infeasible. At present, several measures that do not adversely affect the environment (i.e., green processes) are attractive because they seek to replace the use of unsustainable assets with that of agrarian waste. The waste produced by the agricultural industry is a source of rich protein, and effective techniques to obtain naturally dynamic mixtures from it (primarily using protein hydrolysates) have been developed^3^. Many recent studies have focused on the functional characteristics of low-cost protein sources for the extraction of food ingredients and nutritional supplements. Examples include microalgae ^4^, pigeon pea (*Cajanus cajan*)^5^, wheat bran^6^, peas (*Pisum sativum* L.)^7^, *Pinus halepensis*^8^, and cottonseed^9^.

Rice is a staple food for much of the global population^10^. It is rich in not only energy but also nutrients, minerals, and phenolic cancer prevention agents. Rice is the primary grain developed in Taiwan, with an annual yield of almost 2 million Mg^11^. Broken rice represents about 15% of ground rice. Broken rice is a byproduct of the rice harvesting process. This type of rice is typically utilized in animal feed rather than in human food, despite its low production cost. Broken rice is an inescapable byproduct of rice processing measures. It contains approximately 78–80% starch and 7–8% protein. In contrast to that of whole rice, its market value is considerably lower; therefore, it may be utilized to separate protein from starch. Furthermore, the primary rice protein does not contain gliadin, which is an exceptionally nutritious protein that is found in grains and causes hypersensitive gastrointestinal celiac infection^12^.

Enzymatic hydrolysis is a mild reaction that provides free amino acids and peptides of various sizes, and it can minimize side effects and increase the extraction rate. Enzymatic protein hydrolysate has effective functional and nutritional properties and can be used in dietary protein supplements and clinical treatment^13^. In particular, several vegetable protein hydrolysates obtained by enzymatic hydrolysis, such as soybeans, corn gluten, rice, rice bran, and potatoes, reportedly possess biological benefits, including antihypertensive, antioxidant, antithrombosis and cholesterol-lowering properties^14^. Being a rich source of biologically active substances, plant protein hydrolysates can be used as an alternative source of protein and health-promoting ingredients in specific nutritional products. According to reports, amino acids, such as cysteine, histidine, tryptophan, lysine, arginine, leucine, valine, β-hydroxytryptophan, and their derivatives, exhibit antioxidant activity^15^. In addition, some low molecular weight (MW) peptides containing histidine, tryptophan, and tyrosine also exhibit unique antioxidant properties^16^.

Rice protein hydrolysates (RPH) have attracted attention because rice is the principal food crop for more than half of the world population^17^. However, the effects of MW on RPH antioxidant activity have garnered less exposure. In this study, rice protein isolate was hydrolyzed by using protease enzymatic processes and fractionated into peptides of various MWs to compare antioxidant activities within different in vitro antioxidant evaluation systems.

## Results and discussion

### Extraction yield

Specific and nonspecific proteases were utilized in proteolysis to generate biologically active peptides^18^. As detailed in Table 1, the percentage DH of RPH was 9.4% in this study. Higher DH values could be obtained for the Alcalase protease used in this study compared with those obtained in the literature using hydrolyzed zein^19^, pumpkin oil cake protein isolate ^20^, and canola protein^21^. However, several protein substrates, such as peanut^22^ and corn protein^19^, had similar DH to the RPH examined in this study. Such behaviors may be related to reduced peptide bonds that can be cleaved, competition between the substrate and hydrolysate, and enzyme denaturation to reduce its activity^23^.

**Table 1:** Degree of hydrolysis (DH), yield, and protein content (PC) of rice protein hydrolysate fractions.

Peptide sizes	DH (%)	Yield (%)*	PC (%)^#^
Hydrolysate	9.4 ± 0.12	9.7 ± 0.34	3.5 ± 0.05
< 1 kDa	–	4.6 ± 0.21	2.2 ± 0.17
1–10 kDa	–	2.8 ± 0.76	0.4 ± 0.19
> 10 kDa	–	2.4 ± 0.56	0.1 ± 0.12

*Weight ratio of the hydrolysate in the starting material (crude rice protein isolate).

^#^Weight ratio of the protein in the hydrolysate.

The protein content in the RPH solution was 3.5%, and the yield of RPH was 9.7%. The Alcalase protease has wide specificity for peptide bonds during hydrolysis^24^. The yield of different RPH fractions is between 2.4 and 4.6%, and the low MW (LMW) fraction (< 1 kDa) exhibited the highest yield, which indicates a high content of small peptides in the protein hydrolysate. A similar trend was reported for high levels of LMW peptides in canola protein hydrolysate^25^. The RPH fractions’ protein content was between 0.1% and 2.2%, and it decreased with MW.

### Activity of antioxidants

**Figure 1:**
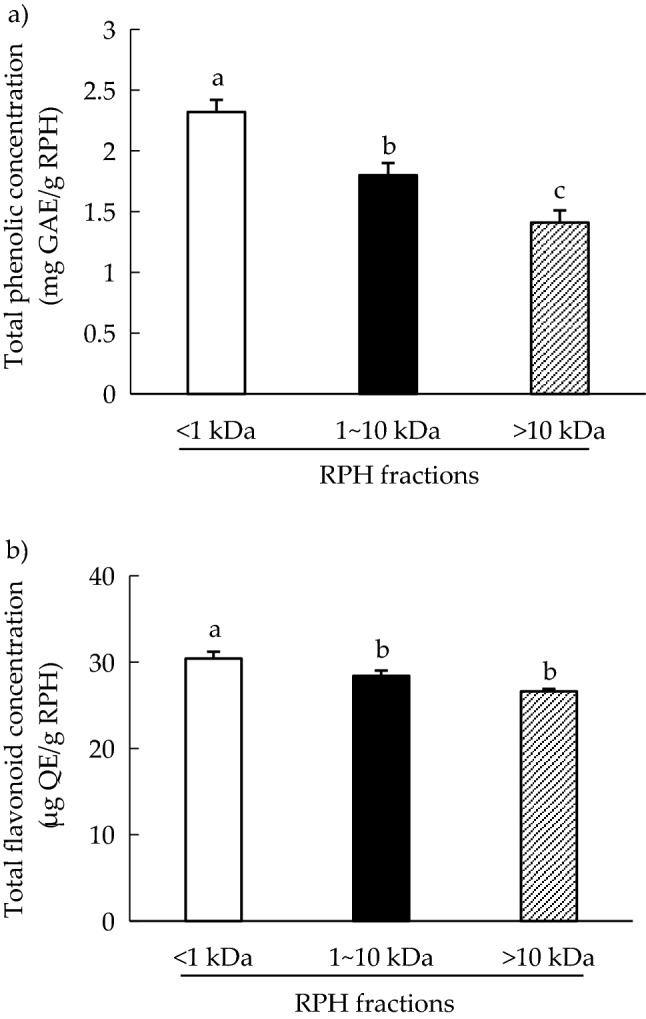
(**a**) Total phenolic concentration and (**b**) total flavonoid concentration of rice protein hydrolysate (RPH) fractions. Means denoted by different letters in the bar charts differ significantly with each other (*p* < 0.05).

*TPC and TFC.* The TPC of RPH samples was obtained by inputting the samples’ optical absorbance value into the gallic acid calibration curve. A higher absorbance indicated a higher TPC. Figure 1a indicates that the TPCs were 2.3 ± 0.72, 1.8 ± 0.52, and 1.4 ± 0.28 mg GAE/g RPH for the RPH fractions of MW < 1 kDa, MW 1–10 kDa, and MW > 10 kDa, respectively. The TFC content was obtained using a similar procedure as that used for TPC. Figure 1b reveals that RPH fractions had a TFC of 26.6–30.4 µg QE/g RPH, and the MW < 1 kDa fraction had the highest TFC among the three fractions (*p* < 0.05). The TPC of RPH samples may contain hydroxybenzoic acids (gallic acid, p-hydroxy benzoic acid, protocatechuic acid, vanillic acid, syringic acid) and hydroxycinnamic acid (caffeic acid, chlorogenic acid, cinnamic acid, ferulic acid, p-coumeric acid, sinapic acid) and TFC may be from apigenin, catechin, kaempferol, luteolin, myricetin^26,27^.

#### DPPH free radical scavenging activity

**Figure 2:**
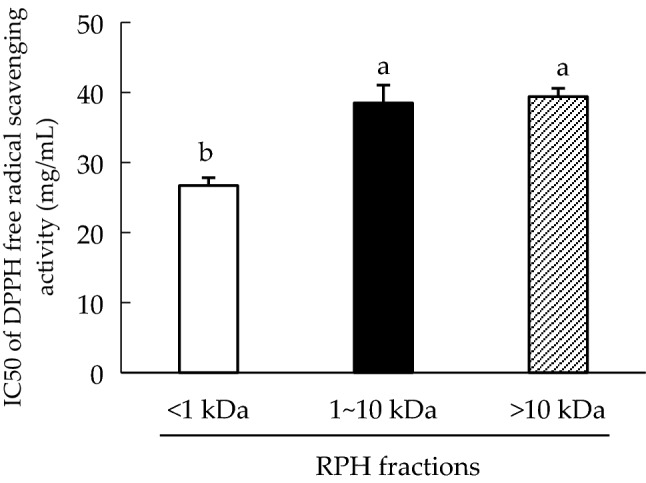
IC50 of 1,1-diphenyl-2-picrylhydrazyl (DPPH) radical scavenging activity of rice protein hydrolysate (RPH) fractions. Means denoted by different letters in the bar chart significantly differ from each other (*p* < 0.05).

Figure 2 displays the IC50 of RPH fractions ranging between 26.7 and 39.4 mg/mL. LMW fractions of RPH fractions (IC50 26.7 ± 1.13 mg/mL) were more significant (*p* < 0.05) than the higher MW (HMW) fractions (IC50 value of 39.4 ± 1.19 mg/mL). LMW peptides exhibited high antioxidant activity, as previously recorded with hemp protein fraction and barley hordein hydrolysate^28,29^.

#### ABTS free radical scavenging activity

**Figure 3:**
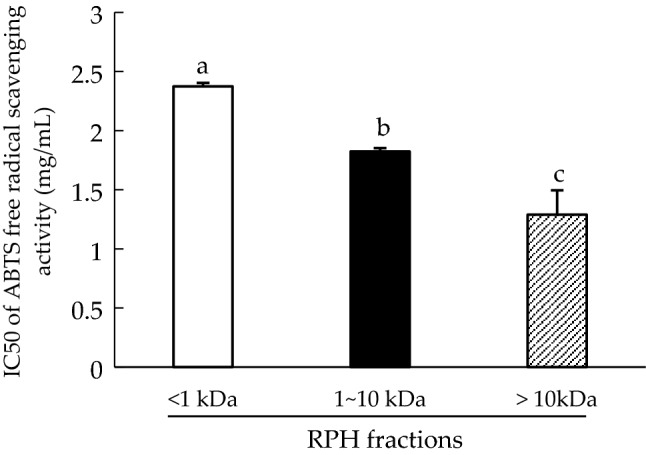
IC50 of 2,2’-azino-bis(3-ethylbenzothiazoline-6-sulfonic acid) (ABTS) radical scavenging activity of rice protein hydrolysate (RPH) fractions. Means denoted by different letters in the bar charts significantly differ with each other (*p* < 0.05).

Figure 3 illustrates the IC50 values of RPH fractions involved with scavenging ABTS free radicals. The results indicated that the MW > 10 kDa fraction (IC50 = 1.29 ± 0.20 mg/mL) is the most effective (*p* < 0.05) among the three fractions. The higher MW fraction had a greater ability to trap ABTS free radicals than the lower one did. This finding is consistent with results of a report on rice bran protein hydrolysates^30^, which suggested that the ABTS free radical scavenging activity of the fraction increased with MWs smaller than 5 kDa.

#### Reducing power

**Figure 4:**
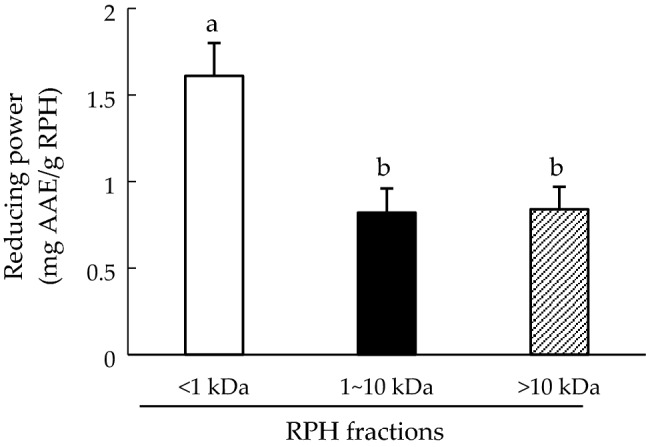
Reducing power of rice protein hydrolysate (RPH) fractions. Means denoted by different letters in the bar charts significantly differ with each other (*p* < 0.05).

The iron reduction activity of peptides was positively correlated with its antioxidant activity. Figure 4 depicts the iron reduction ability of the RPH fractions. The highest iron reduction capacity (1.6 ± 0.19 mg AAE/g RPH) was found in the MW < 1 kDa fraction, which was significantly higher than that of the 1–10 kDa fraction and the MW > 10 kDa fraction (*p* < 0.05). Amino acid components can play a critical role in the reducing activity of peptide fractions. For example, higher glutamic acid, aspartic acid, and glycine content can lead to higher reducing activity^31^, which is consistent with the results of this study.

#### Oxygen radical absorbance capacity (ORAC)

**Figure 5:**
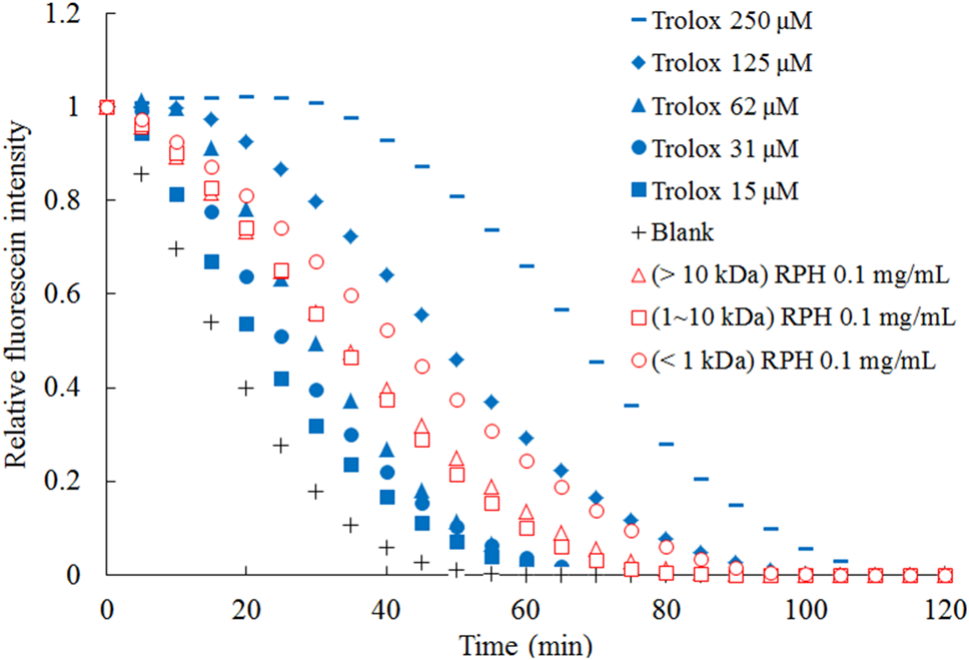
Fluorescence decay curves induced by 2,2'-Azobis(2-amidinopropane) dihydrochloride in the presence of rice protein hydrolysate (RPH) fractions at different concentrations.

Figure 5 represents the dynamic fluorescence decay curves in the ORAC analysis. Computed using the area under the curve, the ORAC values were 774, 576, and 603 µmol TE/g RPH for MW < 1 kDa, MW 1–10 kDa, and MW > 10 kDa, respectively. This trend is similar to the result for reduction capacity, and the fraction with MW < 1 kDa registered a higher ORAC. This finding is consistent with that of Wattanasiritham et al., who reported that the MW of the peptide composed of 6–21 amino acids in the rice bran protein hydrolysate ranged from 0.7 to 1.5 kDa and that the main antioxidant derived from the MW of food protein peptides ranged from 0.5 to 1.8 kDa^32^. The small peptides can easily migrate to react with the target substance; therefore, the LMW fraction exhibited the best antioxidant activity. A similar result was observed in the hydrolysate of sandfish protein and in the protein hydrolysate of Chinese cherry seeds^33,34^. The higher antioxidant activity of the LMW fraction is considered to be due to the easier reaction with free radicals and the more effective elimination of free radicals^35^. Table 2 summarizes the results for antioxidant ability.

**Table 2:** Antioxidant activity of rice protein hydrolysate (RPH) fractions.

RPH fraction	IC50 of DPPH (mg/mL)	IC50 of ABTS (mg/mL)	Reducing power (mg AAE/g)	ORAC (μmol TE/g)
< 1 kDa	26.7 ± 1.13	2.37 ± 0.02	1.6 ± 0.19	774
1–10 kDa	38.5 ± 2.54	1.82 ± 0.02	0.8 ± 0.14	576
> 10 kDa	39.4 ± 1.19	1.29 ± 0.20	0.8 ± 0.13	603

IC50: Half maximal inhibitory concentration.

### Amino acid profiles and MWs of RPH

Table 3 lists the composition percentages and amino acid profiles of RPH fractions. Total amino acids of each RPH fraction were analyzed. High contents of Glu, Asp, Arg, Leu, Tyr, Phe, Ala, and Ser were observed. The predominant type of amino acid was hydrophobic amino acid (HAA), and the content ratio was similar in each fraction. In particular, MW < 1 kDa had the highest content of total hydrophobic amino acids. The proportion of aromatic amino acids (AAA) in the MW < 1 kDa fraction was also the highest. These results accord with those in the literature that a similar amino acid composition is present in the same fraction of mung bean meal protein hydrolysates^36^.

**Table 3:** Amino acid composition of rice protein hydrolysate fractions.

Group	Amino acid^1^	Content (mg/g protein hydrolysate)		
		< 1 kDa	1–10 kDa	> 10 kDa
Acid (−)	Asp	76.4	13.6	5.8
	Glu	142.1	22.4	9.6
Basic ( +)	Lys	34.8	5.0	2.2
	Arg	75.2	10.0	4.5
	His	20.5	2.8	1.2
	Gly	37.8	6.1	2.5
	Ala	52.1	7.6	3.1
	Val	49.5	7.7	3.2
	Ile	34.5	5.2	2.2
	Leu	74.6	10.4	4.4
	Pro	32.8	5.9	2.5
	Met	7.2	0.6	0.6
	Cys	6.8	1.6	0.7
Aromatic	Tyr	50.6	6.7	2.9
	Phe	48.0	6.6	2.9
	Trp*	–	–	–
Hydrophillic	Ser	47.6	7.0	2.8
	Thr	31.6	5.3	2.0
HAA^2^		272.6	39.7	17.2
AAA^2^		98.6	13.3	5.8
BCAA^2^		158.53	23.33	9.88
EAA^2^		300.56	43.69	18.81

*Trp (tryptophan) was not determined.

^1^Asp (aspartic acid); Glu (glutamic acid); Lys (lysine); Arg (arginine); His (histidine); Gly (glycine); Ala (alanine); Val (valine); Ile (isoleucine); Leu (leucine); Pro (proline); Met (methionine); Cys (cysteine); Tyr (tyrosine); Phe (phenylalanine); Trp (tryptophan); Ser (serine).

^2^Hydrophobic amino acids (HAA) = alanine, valine, isoleucine, leucine, methionine, cysteine, and phenylalanine; aromatic amino acids (AAA) = phenylalanine, tryptophan, and tyrosine; branched chain amino acids (BCAA) = leucine, isoleucine, and valine; essential amino acids (EAA) = phenylalanine, valine, tryptophan, threonine, isoleucine, methionine, histidine, leucine, and lysine.

Some reports found that TPC and TFC increased significantly through fermentation or hydrolysis of enzyme treatment ^37–39^. Moreover, protein–polyphenol interaction is not only particularly affected by polyphenol structure but also by surface properties of proteins. Different proteins possess varying amino acid composition, hydrophobicity, and isoelectric point, which influence the binding capacity of proteins with polyphenols^40^. Bohin et al. reported that proline content on protein surface also determine the binding capacity between proteins and polyphenols^41^. In particular, MW < 1 kDa had the highest content of proline in this study. The fraction with MW < 1 kDa contains higher polyphenols and has a high degree of antioxidant capacity.This higher capacity is often described as due to the more open and extended conformation of the peptide chain, usually imposed by proline residues. Polyphenols have a higher ability to bind proline-rich peptides (PRP), although not specifically to proline residues^42^. In fact, this higher affinity for PRP occur in biological proteins such as salivary PRP as well as food proteins such as gliadin^43^. Several structural features in peptide sequencing have been highlighted as affecting their biological function and PRP are the most active^42,44^.

Previous reports^45,46^ showed that different amino acids have different antioxidant activities. Hydrophobic amino acids are known to have strong free radical scavenging activities^45^. Besides, negatively charged acidic amino acids such as glutamic acid and aspartic acid could have strong interaction with free radicals and metal cations. By transferring electrons, alanine, leucine, proline, tryptophan, phenylalanine, tyrosine, and histidine can have strong free radical scavenging activities^46^.

**Figure 6:**
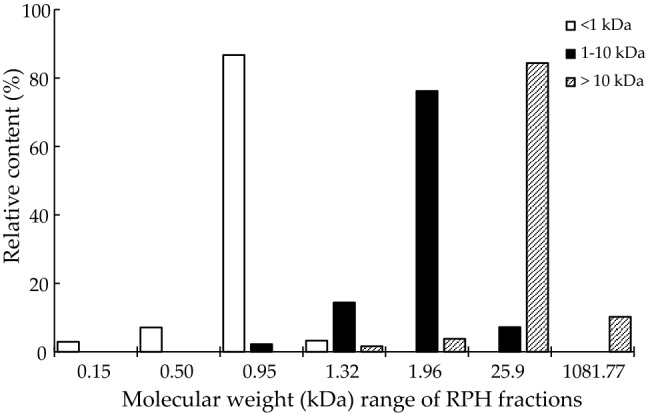
The relative content (%) of different molecular weights (kDa) in rice protein hydrolysate (RPH) fractions.

The cleavage of proteins during enzymatic hydrolysis results in the production of peptides with different MWs (Fig. 6). The MWs of RPH were determined to be in the range of 0.15–1081.77 kDa in a HPLC analysis. RPH fractions exhibited main low and medium MWs of 0.95, 1.96, and 25.9 kDa. The results indicate that effective enzymatic hydrolysis hydrolyzed the protein into peptides with MWs of almost less than 5 kDa. The relative abundance of LMW fractions was related to the results of membrane fractionation. Among the three fractions, the MW < 1 kDa fraction had the highest yields (Table 1). The hydrolysate of protease had a MW of 0.95 kDa, which can be attributed to the wide specificity and efficiency of the protease. He et al. reported similar results for alkaline protease hydrolysate of rapeseed protein^47^, but Makinen et al. reported that 72% of peptides in Alcalase rapeseed hydrolysate were in the range of 1–5 kDa^48^. Other source of proteins or different enzymes used may provide a different trend of result, especially the bioactivities.

The correlation coefficients associated with different tests, including those of amino acid contents, TPC, TFC, and antioxidant activities in UF fractions are summarized in Table 4. A Pearson correlation test indicated that ORAC was significantly and positively correlated with TPC (r = 0.999, *p* < 0.05). The IC50 of radical scavenging activities for DPPH and ABTS were negatively corelated (r =  − 0.986 to − 1.000) with amino acid content for the fractions. The aminoalkanoic acid type in peptide sequences is a crucial factor in determining bioactivity. Total AAA, including tyrosine and phenylalanine, was significantly correlated with reducing power (r = 0.997, *p* < 0.05). The presence of AAA within the peptide sequence of walnut (*Juglans regia* L.) protein hydrolysate greatly influenced its antioxidant activity^49^. Ahn et al. also reported that peptides with 40% phenylalanine in Phe–Phe–Leu–Leu–His sequence enhanced antioxidant activity^50^. Tyrosine and phenylalanine positively affected radical scavenging and acted as a robust proton donor for reaction with electron-deficient radicals^51^. Abundant hydrophobic amino acids (HAAs; specifically, glycine, proline, alanine, valine, leucine, and isoleucine) are the key factors in effective antioxidant activity^52^. The high content of HAA in the LMW fraction, relative to other fractions, may explain its higher antioxidant activity.

**Table 4:** Correlation coefficients of amino acid contents, total phenolic concentration (TPC), total flavonoid content (TFC), and antioxidant activities.

	Amino acid contents			Antioxidant		Antioxidant activities			
	HAA^#^	AAA^#^	BCAA^#^	TPC	TFC	DPPH^1^	ABTS^1^	RP^2^	ORAC
HAA^#^	1	–	–	–	–	–	–	–	–
AAA^#^	1.000**	1	–	–	–	–	–	–	–
BCAA^#^	1.000**	1.000**	1	–	–	–	–	–	–
TPC	0.935	0.933	0.936	1	–	–	–	–	–
TFC	0.916	0.913	0.917	0.999*	1	–	–	–	–
DPPH^1^	−1.000*	−1.000**	−1.000*	−0.929	−0.909	1	–	–	–
ABTS^1^	−0.987	−0.986	−0.987	−0.980	−0.969	0.984	1	–	–
RP^2^	0.997	0.997*	0.997	0.904	0.881	−0.998*	−0.971	1	–
ORAC	0.950	0.948	0.951	0.999*	0.995	−0.945	−0.988	0.922	1

^#^Hydrophobic amino acids (HAA) = alanine, valine, isoleucine, leucine, methionine, cysteine, and phenylalanine; aromatic amino acids (AAA) = phenylalanine, tryptophan, and tyrosine; branched chain amino acids (BCAA) = leucine, isoleucine, and valine;

^1^IC50 value.

^2^RP: Reducing power.

*,**Significant at *p* < 0.05 and 0.01, respectively.

This study showed the antioxidant capacity of RPH was affected by the molecular weight size of protein. Due to steric hindrances, the HMW fraction may have limited access to the centre active structure to react with the target substance. Therefore, LMW fraction easily reacted with radicals and had more potent antioxidant activity^53,54^.

## Conclusions

This study examined the effect of hydrolysate fraction in rice protein. The presence of phenolic compounds in the protein fractions increased the activity of protein extracts. Although the RPH already exhibited significant bioactivity, the protein hydrolysate fraction evaluations indicated that various antioxidant activities significantly decreased with MW. Peptides with a smaller MW and characteristic amino acid sequences possessed higher antioxidant activity (*p* < 0.05). UF selectively fractionated antioxidant peptides, allowing for their activities to be observed. In the future, rice protein hydrolysis fractions can be potentially applied to enhance food formulations, cosmetics, and pharmaceuticals.

## Materials and methods

### Materials

Broken rice (*Oryza sativa*) was obtained from the agricultural experiment station of National Chung Hsing University. Ultrafiltration membranes (1 and 10 kDa molecular weight cut-off [MWCO] sizes) were obtained from Fisher Scientific (Pall Corporation, East Hills, NY, USA). A Masterflex peristaltic pump (Cole Parmer Instrument, Vernon Hills, IL, USA), Swagelok pressure gauge (0Y80 psi; Fluid Mechanics, Queensland, Australia), and ultrapure water (< 0.06 mS) from a Milli-Q purification system were used in this study.

### Enzymatic hydrolysis of rice protein

Rice protein was obtained through precipitation per the method of Chen et al.^45^. In brief, rice protein was enzymatically hydrolyzed with a 2:100 (v/v) enzyme-to-substrate ratio to obtain hydrolysates. Protease (Alcalase, EC 3.4.21.62, serine endo-peptidase, 2.4 AU-A/g; Healthmate, Changhua, Taiwan) was used and the hydrolysis conditions involved setting pH 8.0 at 50 ± 5 °C. After a 4-h reaction period, the protease was inactivated through heating at 85 °C for 15 min. After centrifugation (5000 rpm, 4 °C) for 15 min, the supernatant was lyophilized and stored at − 20 °C before use.

### Determination of degree of hydrolysis

The degree of hydrolysis (DH) was estimated by formal titration according to Taylor^55^. In summary, the pH of the hydrolysate solution (3 wt%) was adjusted to 7.0 using 0.1 M NaOH solution. Formaldehyde solution (37% [v/v]; 10 mL) was further added and stored at room temperature for 5 min, and titration with 0.1 N NaOH solution was then applied until pH 8.5 was reached. The required volume of NaOH solutions was recorded. Total nitrogen (TN) in the sample was determined using the Kjeldahl method^56^. The content of free amino groups and DH were calculated as follows:

1 $${\text{ Free amino group }}\% \, = \, \left[ {{\text{A }} \times {\text{ F }} \times { 14}.00{7 }/{\text{ C }} \times { 1}000} \right] \, \times { 1}00\%$$

2 $${\text{ DH }} = \, \left[ {{\text{free amino group }}\% \, /{\text{ TN }}\% } \right] \, \times { 1}00\%$$

where A is the NaOH solution titer (mL), F is the correction factor, and C is the sample amount (g).

### Protein content in RPH

Protein concentration of water extracts were determined by Biuret method using bovine serum albumin (Sigma Chemical Co, St. Louis, MO, USA) as a standard^57^.

### Ultrafiltration fractionation of RPH

RPH was fractionated by ultrafiltration using 1- and 10-kDa-MWCO membranes. RPH was divided into three fractions: MW > 10 kDa, 1 < MW < 10 kDa, and MW < 1 kDa. The collected three fractions were lyophilized and stored at − 20 °C for further antioxidant assays^58^.

### Determination of amino acid profile

The amino acid composition of rice protein was analyzed using an automatic amino acid analyzer (L-8900; Hitachi, Tokyo, Japan). The RPH sample was sealed in a tube and hydrolyzed with 4 N methanesulfonic acid (115 °C, 24 h). Then, the sample was centrifuged at 12,000 × *g* for 10 min, and the supernatant was collected to determine the amino acid composition. Before injection, the analysis sample was filtered using a 0.22 μm film ^45^. The protein content of RPH was measured using the modified Lowry method^59^.

### Determination of MW distribution

The MW distribution of RPH was determined using a high-performance liquid chromatography system (HPLC; Hitachi, Tokyo, Japan) equipped with a BioSep-SEC-s2000 (size: 300 × 7.8 mm^2^). The optimal conditions for HPLC in the size exclusion mode were determined for the measurement of the MW distribution of RPH samples. An elution of 1 mL, 5 mg/mL sample solution in a column was performed at a flow rate of 0.5 mL/min at room temperature. Peptide size was measured using the calibration curve of standard compounds, including the column performance check standard (comprising bovine thyroglobulin [670 kDa], immunoglobulin A [300 kDa], immunoglobulin G [150 kDa], ovalbumin [44 kDa], and myoblobulin [17 kDa]) and vitamin B12 (1.35 kDa).

### In vitro activities of RPH

All the in vitro activities of RPH solution were determined using the collected protein solutions from ultrafiltration.

#### Total phenolic concentration

The Folin–Ciocalteu technique for determining the total phenolic concentration (TPC) of RPH fractions was utilized^60^. Initially, 200 μL of Folin–Ciocalteu's phenol reagent (0.5 N) was consistently blended through 5-min shaking with 200 μL of RPH; 400 μL of deionized (DI) water and 200 μL of 10% sodium carbonate were also added. The blended solution underwent 60 min of brooding in the dark at room temperature. It was then centrifuged for 15 min at 3000 rpm, and 100 μL of supernatant was used for analysis. To determine the TPC of the gallic acid equivalents (GAE) per gram of dry RPH (mg GAE/g RPH), the optical absorbance information was obtained with reference to a standard curve for gallic acid. The absorbance was acquired at 700 nm through an Epoch Microplate Spectrophotometer (BioTek, VT, USA).

#### Total flavonoid content

Total flavonoid content (TFC) was obtained per the method of Wathoni et al.^61^, with minor modifications. Initially, every one of the 500-μL samples and 2% aluminum chloride were blended together and stored for 10 min. The absorbance at 415 nm was assessed. The outcome was recorded in micrograms of quercetin equivalents (QE) per gram of the dry RPH (µg QE/g RPH).

#### Free radical scavenging activity for 1,1-diphenyl-2-picrylhydrazyl

The method of Tsai et al.^62^ was applied to measure the free radical scavenging activity (specifically, 1,1-diphenyl-2-picrylhydrazyl [DPPH]) of protein hydrolysates. RPH solution (0.5 μL) was added to 50 μL of DPPH (198 μM) in a 99.7% ethanol solution held in a 96-well plate. The plate was vortexed for 10 s and then left to stand for 30 min in the dark at room temperature. The absorbance of the solution was measured at 517 nm using the Epoch Microplate Spectrophotometer.

$${\text{DPPH}}\cdot\left( {{\text{deep purple}}} \right) \, + {\text{ AH}}\rightarrow {\text{DPPH-H }}\left( {{\text{pale yellow}}} \right) \, + {\text{ A}\cdot}$$

#### Free radical scavenging activity for 2,2’-azino-bis(3-ethylbenzothiazoline-6-sulfonic acid)

The 2,2’-azino-bis(3-ethylbenzothiazoline-6-sulfonic acid) (ABTS) radical scavenging activity of RPH fractions was determined according to the procedure of Wu et al.^63^. In brief, the ABTS free radicals were produced from the reaction of 7 mM ABTS reagents in H_2_O and 2.45 mM potassium persulfate, stored in the dark at 4 °C for 16 h. The various concentrations of RPH fractions were mixed with 180-μL ABTS free radicals and then maintained for 10 min in the dark at room temperature. The absorbance of the final mixture was measured at 734 nm.

$${\text{ABTS}}^{ +}\cdot \left( {{\text{blue-green}}} \right) \, + {\text{ AH}}\rightarrow {\text{ABTS }}\left( {\text{pale blue}} \right) \, + {\text{ H}}^{ + } + {\text{ A}\cdot}$$

#### Reducing power

The reducing power of canola protein hydrolysates was measured according to Lin et al.^64^, with some modifications. Briefly, RPH samples were dissolved in a 0.2 M phosphate buffer (pH 6.6). An aliquot (200 μL) of sample solution was then added to 100 μL of a 1% potassium ferricyanide solution and incubated at 50 °C for 20 min. After incubation, DI water (400 μL) and a ferric chloride solution (0.1%, 100 μL) were added. The absorbance of the reaction mixture was then recorded immediately at 700 nm. A high absorbance indicated a high reducing power. A control without any hydrolysates and a blank containing only hydrolysate samples were used in experiments. The reducing power was reported in terms of micrograms of ascorbic acid equivalents per gram of dry RPH sample (mg AAE/g RPH).

$${\text{K}}_{{3}} {\text{Fe}}\left( {{\text{CN}}} \right)_{{6}}\left( {{\text{yellow}}}\right)\ + {\text{ Antioxidant}}\rightarrow {\text{K}}_{{4}} {\text{Fe}}\left( {{\text{CN}}} \right)_{{6}} + {\text{ Antioxidant-}}{\mathrm{oxide}}$$

$${\text{3K}}_{{4}} {\text{Fe}}\left( {{\text{CN}}} \right)_{{6}} + {\text{ 4Fe}}^{{{3} + }} \rightarrow {\text{Fe}}_{{4}} \left[ {{\text{Fe}}\left( {{\text{CN}}} \right)_{{6}} } \right]_{{3}}\left( {{\text{dark blue}}} \right) \ + {\text{ 12K}}^{ + }$$

#### Oxygen radical absorbance capacity

An oxygen radical absorbance capacity (ORAC) assay was conducted per the method in the literature^65^. Briefly, 100 μL of fluorescein (10 μM) and a 50 μL sample (0.1 mg/mL) of Trolox (15–250 μM) were transferred into a 96-well plate. Subsequently, 50 μL of 2, 2’-azobis(2-methylpropionamidine) dihydrochloride (500 mM) was added and the fluorescence of the mixture was recorded for 2 h at 485-nm excitation and 528-nm emission in a Synergy LX microplate reader (BioTek, Winooski, VT, USA). The ORAC was reported in terms of micromoles of Trolox equivalents (TE) per gram of dry RPH sample (µmol TE/g RPH)^65^.

$${\text{ROO}}\cdot + {\text{ AH}} \to {\text{ROOH}} + {\text{A}\cdot}$$

$${\text{ROO}}\cdot + {\text{ FLH}} \to {\text{ROOH}} + {\text{FL}\cdot}$$

### Statistical analysis

All experiments were performed in greater than triplicate. Data were recorded as mean ± standard deviation (SD), and statistical analysis using one-way analysis of variance and Duncan's post hoc test were executed in SPSS (version 20.0; SPSS, Armonk, NY, USA). Statistical significance was indicated by *p* < 0.05. A Pearson correlation test was performed to highlight the correlation coefficients among the means.

### Ethical approval

Oryza sativa plants were used in this study. Oryza sativa was obtained from the Agricultural Experiment Station, College of Agriculture and Natural Resources, National Chung-Hsing University (Taichung, Taiwan) were kindly provided by Dr. Wang Chang-Sheng (Taichung, Taiwan). The experimental research on plants complied with the relevant institutional, national, and international guidelines and legislation.
